# Construction and empirical analysis of the ideological and political teaching ability inspection model of professional teachers in universities empowered by digital intelligence

**DOI:** 10.3389/fpsyg.2025.1550797

**Published:** 2025-09-24

**Authors:** Xueying Yang, Wenxin Ye, Jia Wei, Huanyang Yu

**Affiliations:** ^1^School of Economics and Management, Changchun University of Technology, Changchun, China; ^2^School of Materials Science and Engineering, Jilin Jianzhu University, Changchun, China

**Keywords:** college professional teachers, digital intelligence empowerment, ideological teaching ability, political teaching ability, inspection model

## Abstract

The development of digitalization, big data, and information technology has profoundly affected higher education. College and university teachers operate within a new innovative ecology, encompassing everything from the acquisition of information resources to the presentation of teaching methods across space, time, patterns, content, and pedagogy. Under the concept of comprehensive digital education, the efficient integration of curriculum expertise and professional education, supported by digital technology can enhance the digital ability and proficiency of professional course instructors in higher education and improve the goal of human education. To this end, the digital intelligence-empowered inspection model for college professional teachers’ ideological and political teaching ability inspection is constructed to enable them to diagnose and assess their own ideological and political teaching capacity and to plan the direction of their development and enhancement. Colleges, universities, and management organizations can intuitively understand the performance levels and training needs related to professional teachers’ ideological and political teaching ability, allowing them to improve this ability in a targeted and practical way.

## 1 Introduction

The digitization of education has become a global phenomenon, as schools and educational institutions increasingly integrate technology into teaching and learning processes (Bygstad et al., 2022). The pandemic has accelerated this shift, highlighting the importance of digital tools for distance and online learning (Akour and Alenezi, 2022). Teachers not only deliver content but also serve as facilitators of technology-mediated learning experiences (Tatto, 2023). They must adapt to various digital tools, platforms, and pedagogical approaches to effectively engage students (Rawal, 2024; Wang, 2024). Teachers’ adaptability and digital competence influence students’ learning outcomes. Those who continuously update their skills are better equipped to meet the diverse needs of digitally savvy students (Väisänen and Hirsto, 2020).

In the context of the “Internet +” era, the use of Internet technologies and platforms for ideological and political education can greatly expand the temporal and spatial boundaries of education and enable a hybrid teaching mode combining online and offline modes (Luo, 2020). For example, through online discussion platforms, students can express themselves and interact with classmates and teachers at any time, enhancing interactivity and participation in ideological and political education (Wang et al., 2023). In addition, digital technology and artificial intelligence can be utilized to accurately deliver ideological and political education tailored to students’ interests and needs, thereby improving the relevance and effectiveness of education (Freeman et al., 2017).

The influence of digital and intelligent technologies on the thoughts, values, and behaviors of modern college students has been significant, creating a demand for innovation in the instructional methods used to teach them (Watfa and Audi, 2017). A recommendation approach based on collaboration between schools and enterprises has been developed (de Fátima Cruz et al., 2022; Pereira and Franco, 2023). This approach enhances student engagement in political and philosophical thinking.

An ideological and political teaching management system based on the MVC architecture has been constructed for college and university students. Through this system, teachers can better understand students’ learning conditions and address the shortcomings of traditional ideological and political teaching methods (Hongmei and Songlin, 2021). Under the guidance of digital technology, a classroom emotion analysis tool for ideological and political courses has been developed by combining data mining technology, which can analyze students’ emotions in the classroom through multimodal emotion recognition, understand problems in their learning process, and promote the reform of course teaching methods (Honglan and Wu, 2021; Zhao and Yu, 2024).

Discussions on the development of new media technologies and their influence on social lifestyle shows that the widespread application of new media technologies is driving profound reforms in curriculum education for college students. Therefore, colleges and universities should further strengthen the application of new media technologies to integrate them more effectively into classroom teaching and meet students’ personalized learning needs. As times progress, teaching models continue to evolve. College teachers should accelerate the reform and optimization of teaching methods to adapt to changes. To better apply online learning platforms for the education of college students, their online learning behaviors should be monitored to detect abnormalities in their learning behavior (Zhu and Zheng, 2021). A network security information management system, based on artificial intelligence and a data security model, was constructed to ensure the security of teaching systems and improve information management in education to meet the needs of colleges and universities (Zheng et al., 2024).

A comprehensive ideological and political education system was constructed using a wireless network to manage distributed educational resources, significantly improving the information of ideological and political education in higher education institutions. In analyzing the innovative reform of such education in the digital era, a computer simulation system has been used to analyze students’ teaching behaviors. This system aligns with the practical needs of ideological and political education in colleges and universities (Makransky et al., 2019; Jiao et al., 2021). With the passage of time and societal progress, traditional ideological and political education is becoming increasingly ineffective. It is necessary to integrate this form of education into students’ daily lives to meet their psychological needs’. Students must be guided to view, evaluate, and solve problems from a developmental perspective and to form correct attitudes and values (Freeman et al., 2017; Howard, 2017).

Given the current educational background shaped by the top-level design of ideological and political concepts, the organic combination of professional courses with ideological and political instruction has become very important (Wang, 2022). The ideological and political teaching of professional teachers refers to “the teaching of professional courses [that carry] ideological and political education” (Shao, 2023). That is, professional course instructors integrate ideological and political education of students in all aspects of education and teaching and realize the organic unity of subject-specific knowledge transmission with the guidance of ideological, political, and moral values. It is achieved by the creation of a positive and subtle educational and learning atmosphere (Chen, 2023; Moreira et al., 2018). From a pedagogical perspective, college teachers’ ideological and political teaching ability is reflected in their ability to develop and implement curricula (Liu and Jia, 2023). Specialized courses serve as the core platform for both preaching and teaching, functioning as an important channel for leading students into professional fields.

## 2 The construction of the ideological and political teaching ability inspection model of professional teachers in universities empowered by digital intelligence

In the educational context, “digital intelligence” refers to the use of digital tools and methodologies, such as bibliographic coding, behavioral event interviews, the Delphi method, and questionnaire surveys, enhanced by advanced technologies like big data analytics and dynamic AI visualizations. Dynamic data collection, analysis, and feedback can be used to accurately measure and dynamically optimize teachers’ ideological and political teaching capabilities. The construction of this inspection model is grounded in innovation based on critical pedagogy, transformational learning theory, the TPACK framework, and deep learning theory. It establishes five major capability dimensions: ideological and political literacy and cognitive ability, exploration and development ability, organization and design ability, implementation and innovation ability, and evaluation and support ability. Based on an analysis of its reliability and validity, it is believed that enhancing the integration of ideological and political education with professional education in colleges is essential. Digital intelligence in this model functions not only as a technical tool but also as a medium for combining ideological and political education with professional knowledge.

### 2.1 The theoretical basis for building a verification model of ideological and political teaching ability of professional teachers in colleges and universities empowered by digital intelligence

This study constructs a teaching framework supported by four major international educational theories. First, Freire’s “critical pedagogy” establishes a philosophical foundation for integrating ideological and political education. His theory of dialogic education and critical consciousness development (Freire, 1970) requires teachers to reveal the power structures and social implications of subject knowledge through curriculum design. Second, Jack Mezirow’s “transformational learning theory” (Mezirow, 1992) provides a cognitive transformation mechanism for this framework through a three-stage model of “reality critique—meaning reconstruction—practical intervention.” Third, Schultman’s “pedagogical content knowledge” (PCK) and the “TPACK” technology integration framework (Shulman, 1986; Mishra and Koehler, 2006) offer a methodology for interdisciplinary integration, enabling dynamic interaction between technological tools, critical pedagogy, and subject knowledge through GIS technology. Finally, “deep learning theory” (Biggs, 2003) optimizes teaching implementation from a cognitive psychology perspective by stimulating students’ deeper motivation to understand the social significance of knowledge and promoting a shift from surface-level knowledge memorization to critical thinking.

The model integrates the social critique foundation of critical pedagogy, incorporating elements such as political identity, national sentiment, and social responsibility into professional classroom teaching, thereby enhancing the precision and systematic implementation of ideological and political education. Based on the “three-stage model” of transformation learning theory, it constructs precise training pathways by considering teaching experience, title, and subject differences. The model also extends the TPACK framework to ideological and political education for college teachers, emphasizing the deep integration of digital technology with teaching goals. Leveraging the cognitive framework of deep learning theory, it integrates the synergistic mechanism of political attributes and technological governance, guiding professional teachers to leverage digital technologies and current political hotspots and effectively refine the integration and packaging of ideological and political elements with courses. Global trends in value-based and civic education reflect national directives on moral and ideological education, while also balancing political objectives with pedagogical rationality.

### 2.2 Construction and empirical research of the verification model of the ideological and political teaching ability of professional teachers in colleges and universities empowered by digital intelligence

To better understand the current status of ideological and political instruction by professional instructors at digitally intelligent institutions, and to understand how teaching abilities vary across different types of universities, ages, academic qualifications, titles, professional teachers, and their ideological and political training, this study develops a comprehensive evaluation mechanism. The model measures ideological and political teaching ability across the whole teaching process, such as teachers’ own ideological and political literacy, their willingness to integrate ideological and political education into the curriculum, their effectiveness in implementation, their role in cultivating students’ ideological and political understanding, and institutional safeguards that support ideological and political education in the curriculum. Empirical methods such as literature coding, behavioral interviews, the Delphi method, and questionnaire surveys were used to design ability factor and construct the model. The resulting framework enables professional teachers in universities to test and evaluate their own ideological and political teaching ability. It also facilitates targeted evaluation and improvement by identifying individual and institutional gaps in teaching capacity.

#### 2.2.1 Coding of literature

To better understand the influencing factors related to measuring the ideological and political teaching ability of teachers in colleges and universities, we employed literature coding techniques. Using the CKNI database, we conducted literature searches on themes such as ideological and political education in higher education, teaching ability, digitization, big data, etc., and coded the ability, key points, and elemental grades of the literature to develop a preliminary model for evaluating the political and ideological instruction skills of professional educators at institutions equipped with digital intelligence.

#### 2.2.2 Behavioral event interview text coding

Building on the preliminary assessment model of professional instructors’ political and ideological teaching skills in digitally intelligent institutions, we designed the interview outline based on the evaluation index system derived from literature coding. We identified senior-level interviewees and conducted behavioral event interviews. Fifteen professional teachers and ideological and political teachers from key universities in Changchun, Tianjin, and Chongqing participated in interviews on the subject “digital intelligence empowers the ideological and political teaching ability of university teachers.” The interviews were analyzed and refined with the evaluation indexes, elements, grades, etc. and categorized and coded into the initial model of the inspection model for ideological and political teaching ability.

#### 2.2.3 Delphi method

**Figure 1:**
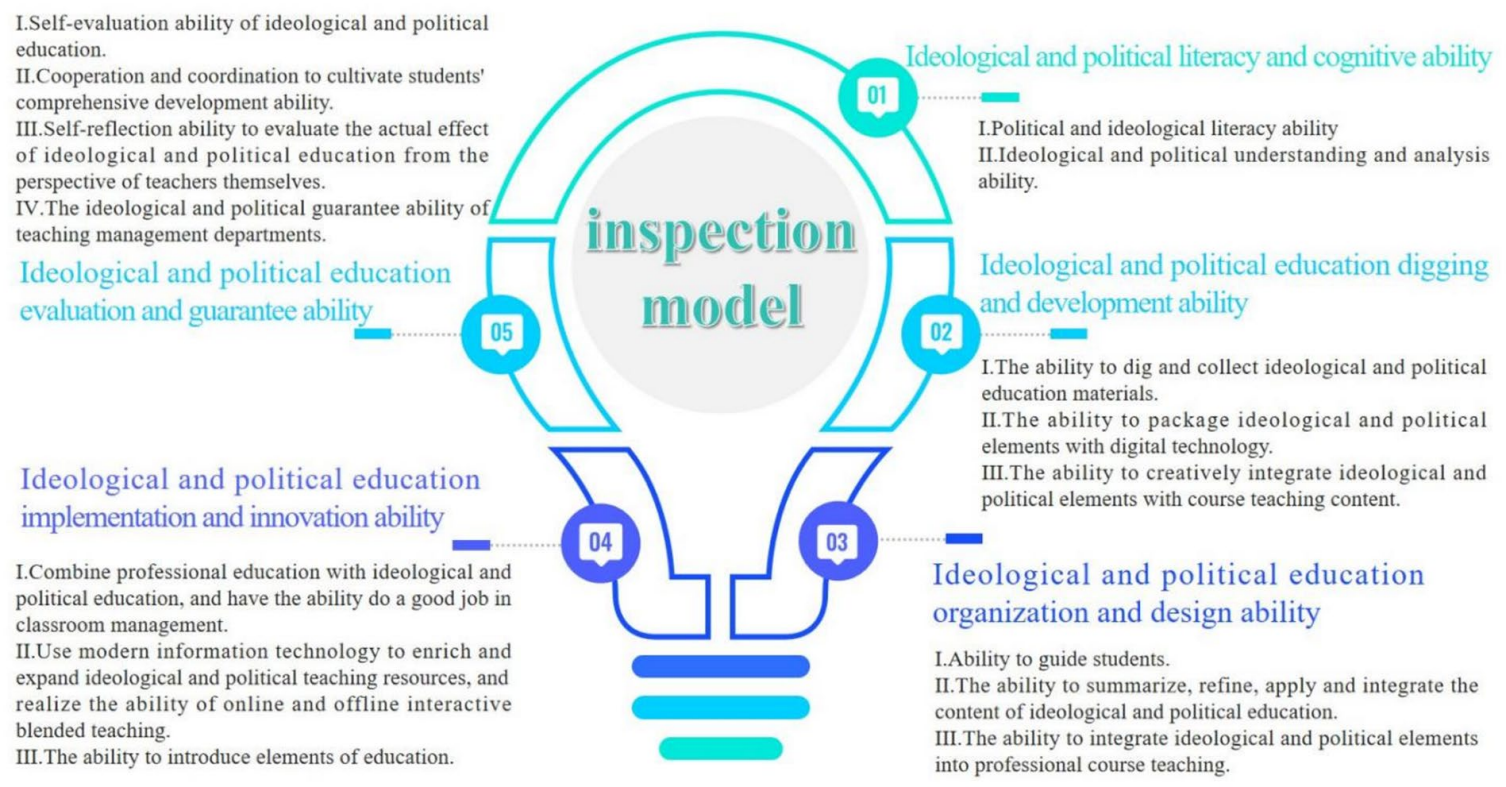
The dimension and sub-component relationship diagram of the competency check model of ideological and political teaching ability of teachers in colleges and universities empowered by digital intelligence.

The Delphi method was further applied to solicit opinions from 12 experts and scholars, specializing in ideological and political education and university teaching, all holding senior professional titles. Based on the evaluation elements, index systems, ability points, ability composition, and hierarchical interpretation, inappropriate indicators were eliminated. After three rounds of feedback adjustment, the finalized evaluation system for the ideological and political teaching ability of professional teachers was finally determined. The system consists of five multi-level indicators: (1) Ideological and political literacy and cognitive ability. (2) Ability of ideological and political mining and development of the course. (3) Ability of ideological and political organization and design of the subject. (4) Ability of ideological and political implementation and innovation of the course. (5) Ability of ideological and political evaluation and guarantee of the course. The components of each competency are further analyzed and shown in Figure 1.

#### 2.2.4 Questionnaires

We selected teachers of professional courses from nine higher education institutions (both key and local universities) across three cities, Changchun, Tianjin, and Chongqing, and conducted a questionnaire survey. A total of 300 questionnaires were distributed; after excluding seven invalid responses, the final dataset included 293 valid questionnaires. The effective recovery rate was 97.7%. SPSS17.0 was used to empirically analyze the valid responses.

The validity and reliability of the model for inspecting the political and ideological teaching skills of professors at universities equipped with digital intelligence were examined through this empirical study. Based on the model’s key competency, we designed a questionnaire consisting of two parts. The first part evaluated the performance level of ideological and political teaching abilities in the context of digital intelligence empowerment. The second part investigated the training needs of these instructors for enhancing such abilities during a digital transformation. Performance was evaluated using four levels: “very poor,” “poorer,” “better,” and “excellent.” For the evaluation of training needs, the four levels were “not needed,” “not too needed,” “more needed,” and “very needed.”

Quantitative methods such as questionnaire-based data collection, factor analysis, reliability and validity testing, and one-way ANOVA were employed to validate the model. Meanwhile, the models were constructed through behavioral event interviews, the Delphi method, and literature coding, through qualitative research. Descriptive analysis was used to describe the current status, training needs, and differences in ideological and political teaching capabilities among professional teachers in higher education. The core objective of this tool validation study was to develop and verify the “Digital Intelligence Empowerment Model for Ideological and Political Education Teaching Capabilities of Professional Teachers in Higher Education Institutions” through model construction, reliability and validity testing, and empirical analysis.

## 3 Construction results of the inspection model for ideological and political teaching ability of professional teachers in universities empowered by digital intelligence

### 3.1 Structural framework of ideological and political teaching ability

A model was developed to assess the ideological and political teaching skills of instructors at institutions utilizing digital intelligence. After passing the reliability and validity testing, a model framework containing five basic elements and 15 key points of capability indicators was established, as shown in Figure 1.

### 3.2 Hierarchical analysis of the inspection model

The 15 factor competency points were categorized in a progressive hierarchy of unqualified, qualified, good, and outstanding. For example, the second factor of the “ability to explore and develop the ideological and political education” is defined as “the ability to effectively package the ideological and political elements with the help of digital technology and current political hotspots.” Based on it, we constructed the list shown in Table 1.

**Table 1:** List of the hierarchical analysis of the ability points of the factor “Ability to effectively package the elements of ideology and politics with the help of digital technology and current political hotspots”.

Ability points	Layer resolution			
	(Unqualified)	(Qualified)	(Good)	(Outstanding)
Integration of ideological and political elements using digital technology and current political hotspots	The development and design of ideological and political elements for courses without combining digital technology and current political hotspots	It has relevant ideological and political elements, and can present them with the help of digital technology.	I can use digital technologies (WeChat, video, etc.) to explore relevant ideological and political elements, explain them reasonably, and combine them with current political hot spots.	I can make full use of digital technology and current political hotspots to effectively and finely package ideological and political elements within course content, guiding students with clear ideological and political values.

### 3.3 Reliability and validity analysis of the model for inspecting the ideological and political teaching ability of professional teachers in universities empowered by digital intelligence

#### 3.3.1 Confidence analysis of the inspection model

##### 3.3.1.1 Confidence analysis of model fundamentals

To determine whether the five fundamental components of the inspection model for professional teachers’ ideological and political teaching abilities in digitally intelligent universities were internally consistent, Cronbach’s alpha value analysis was conducted, and the minimum and maximum Cronbach’s alpha values of the five basic elements were 0.808 and 0.873, respectively. This value exceeds 0.8, indicating strong internal consistency and a high degree of reliability.

##### 3.3.1.2 Overall confidence analysis of the model

Fifteen ability points in the inspection model were tested for internal consistency using the CITC and Cronbach’s alpha values. Its Cronbach’s alpha coefficient was higher than 0.8, its CITC values were all higher than 0.6, and the Cronbach’s alpha values of the deleted items were all lower than 0.954, indicating that the model had a high degree of reliability.

#### 3.3.2 Validity analysis of the inspection model

##### 3.3.2.1 Exploratory factor analysis of the model

KMO and Bart’s sphere tests were performed on the 15 factor ability points in the inspection model, and the results are presented in Table 2.

**Table 2:** List of KMO and Bartlett’s tests for the inspection model of ideological and political teaching ability of teachers in universities empowered by digital intelligence.

KMO and Bartlett		
KMO value	0.950	
Bartlett sphericity test	Approximate chi square	3314.006
	df price	105
	*p* price	0.000

The above table shows that the KMO value was 0.950, with a value > 0.7. The chi-square value of Bartlett’s sphere test was 3314.006, with 105 degrees of freedom and a *p*-value of 0.00, indicating that the research data were suitable for extracting information.

Factor extraction was performed using principal component analysis, resulting in five common factors. The variance explained by values was 24.876, 16.667, 14.539, 12.070, and 11.029%, respectively. The cumulative variance explained after rotation was 79.182% > 50%, which is satisfactory. After utilizing the maximum variance rotation method, a rotated component matrix was obtained, as listed in Table 3.

**Table 3:** List of rotated component matrices of the inspection model of ideological and political teaching ability of professional teachers in universities empowered by digital intelligence.

Ability points	Element				
	1	2	3	4	5
1. Ideological and political literacy ability.	0.264	0.839	0.139	0.224	0.149
2. Ability to understand and analyze ideological and political education.	0.335	0.806	0.164	0.215	0.164
3. Constantly improve teaching methods, innovate teaching concepts, and fully explore and collect ideological and political education materials.	0.294	0.530	0.457	0.002	0.458
4. The ability to effectively package ideological and political elements with the help of digital technology, incorporating current political hot spots.	0.329	0.161	0.785	0.121	0.257
5. The ability to creatively integrate ideological and political education resources and course teaching content.	0.479	0.198	0.538	0.170	0.394
6. The ability to fully understand the students and guide them.	0.804	0.211	0.269	0.143	0.142
7. Ideological and political teaching organization and design ability.	0.742	0.325	0.297	0.171	0.094
8. The ability to integrate political identity, national feelings, social responsibility, and other elements into professional classroom teaching.	0.596	0.283	0.280	0.232	0.308
9. Combine professional education with ideological and political education around the trinity teaching objectives of knowledge transmission, ability training, and value shaping and have the ability to do a good job in classroom management.	0.591	0.330	0.364	0.278	0.179
10. Use modern information technology, enrich and expand the teaching resources of ideological and political education, and use modern information technology to realize the ability of online and offline interactive mixed teaching.	0.203	0.173	0.682	0.583	0.044
11. Introduce the ability of educational elements to realize the interpersonal interaction between teachers and students in class and after class.	0.652	0.216	0.110	0.474	0.194
12. Ideological and political evaluation ability of itself.	0.488	0.354	0.213	0.532	0.311
13. The influence ability of collaborative education has an impact on students’ ideology and cultural literacy, actively participates in various activities, and produces changes in performance, emotion, attitude and behavior.	0.570	0.357	0.151	0.332	0.323
14. Self-reflection ability to evaluate the actual effect of ideological and political education from the perspective of teachers themselves.	0.381	0.293	0.219	0.681	0.295
15. The quality of the teaching management department.	0.220	0.212	0.236	0.261	0.832

From Table 3, all 15 competence points in the inspection model of professional instructors’ ideological and political teaching abilities at universities equipped with digital intelligence had loadings of at least 0.532, with no overlap between components. Corresponding the 5 factors with the points of ability shows that the dimensions of exploratory factor analysis are completely consistent with the dimensions of the inspection model.

#### 3.3.3 Validation factor analysis of the model

To assess the soundness of the inspection model, a five-factor structure based on the 15-item questionnaire measuring professional instructors’ ideological and political teaching abilities in digitally intelligent universities was constructed. The fitting index was used to evaluate fit indices, as shown in Table 4.

**Table 4:** List of 5-factor goodness-of-fit judgments of the inspection model of the ideological and political teaching ability of professional teachers in universities empowered by digital intelligence.

Model	Chi-square degrees of freedom ratio x^2^/df	SRMR	RMSEA	NFI	IFI	CFI	NNFI
5 Factor model	2.968	0.038	0.082	0.93	0.952	0.952	0.937

As shown in Table 4, the 5-factor model, x^2^/df = 2.968 < 3, SRMR = 0.038 < 0.1, RMSEA = 0.1 < 0.1, NFI = 0.93 > 0.9, IFI = 0.952 > 0.9, CFI = 0.952 > 0.9, and NNFI = 0.937 > 0.9, met all standard fit criteria.

To confirm the structural rationality of the model, discriminant and convergent validity were tested, as shown in Table 5. All standardized factor loadings of the competency essentials exceeded 0.5, indicating a strong correlation. The CR values of the essentials were 0.873, 0.833, 0.86, 0.811, and 0.879, which were all greater than 0.8 and above the 0.6 critical value. The AVE values were 0.775, 0.625, 0.672, 0.589, and 0.646, with a minimum value of 0.589, which was greater than the 0.5 critical value. These findings demonstrate that the model possesses high convergence and differentiation validity of the ideological and political teaching capacity of university teachers (Table 5).

**Table 5:** List of the differentiation validity and convergence validity of the ideological and political teaching ability of professional teachers in universities empowered by digital intelligence.

Factor (latent variable)	Measurement items (explicit variables)	Factor loading	CR	AVE
Ideological and political educational literacy and cognitive ability	1. Ideological and political literacy ability.	0.858	0.873	0.775
	2. Ability to understand and analyze ideological and political education.	0.902		
Ideological and political education mining and development ability	3. Constantly improve teaching methods, innovate teaching concepts, and fully explore and collect ideological and political education materials.	0.754	0.833	0.625
	4. With the help of digital technology, current political hot spots, and effective packaging ability for ideological and political elements.	0.78		
	5. The ability to creatively integrate ideological and political education resources and course teaching content.	0.836		
Ideological and political organization and design ability	6. The ability to fully understand the students and guide them.	0.814	0.86	0.672
	7. Ideological and political teaching organization and design.	0.845		
	8. The ability to integrate political identity, national feelings, social responsibility and other elements into professional classroom teaching.	0.8		
Ideological and political education implementation and innovation ability	9. Combine professional education with ideological and political education around the trinity teaching objectives of knowledge transmission, ability training, and value shaping and have the ability to do a good job in classroom management.	0.821	0.811	0.589
	10. Use modern information technology, enrich and expand the teaching resources of ideological and political education, and use modern information technology to realize the ability of online and offline interactive mixed teaching.	0.696		
	11. Introduce the ability of educational elements to realize the interpersonal interaction between teachers and students in class and after class.	0.78		
Ideological and political education evaluation and guarantee ability	12. Ideological and political evaluation ability itself.	0.882	0.879	0.646
	13. The influence ability of collaborative education has an impact on students’ ideology and cultural literacy, actively participates in various activities, and produces changes in performance, emotion, attitude, and behavior.	0.802		
	14. Self-reflection ability to evaluate the actual effect of ideological and political education from the perspective of teachers themselves.	0.831		
	15. The quality of the teaching management department.	0.688		

## 4 Effectiveness analysis of the ideological and political teaching ability inspection model for teachers of specialized courses in universities empowered by digital intelligence

### 4.1 Effectiveness analysis of the ideological and political teaching ability inspection model for college professional teachers empowered by digital intelligence

Colleges and universities, as important institutions for national talent cultivation, ensure that qualified successors are trained in socialism. It has become increasingly important to improve the political literacy, ability level, and ideological and political awareness of the professional teaching team. Organically integrating professional courses with ideological and political education is a necessary ability for every educator. The inspection model for ideological and political teaching ability, empowered by digital intelligence, provides teachers with a clearer understanding of their performance levels.

This model serves as a reference tool for teachers to understand their deficiencies and gaps in comparison to better performing peers regarding their ability to deliver ideology and politics of the curriculum. It encourages self-reflection on areas needing improvement and enables targeted efforts to strengthen these ability points. This model helps college teachers to improve their methods and strategies for ideological and political teaching abilities and accelerate the integration and unification of college curriculum professionalism.

### 4.2 Analysis of the effectiveness of the model for inspection of the ideological and political teaching ability of teachers in universities empowered by digital intelligence by teaching managers and training institutions in universities

#### 4.2.1 The overall evaluation of the performance level of ideological and political teaching ability among professionals empowered by digital intelligence

**Figure 2:**
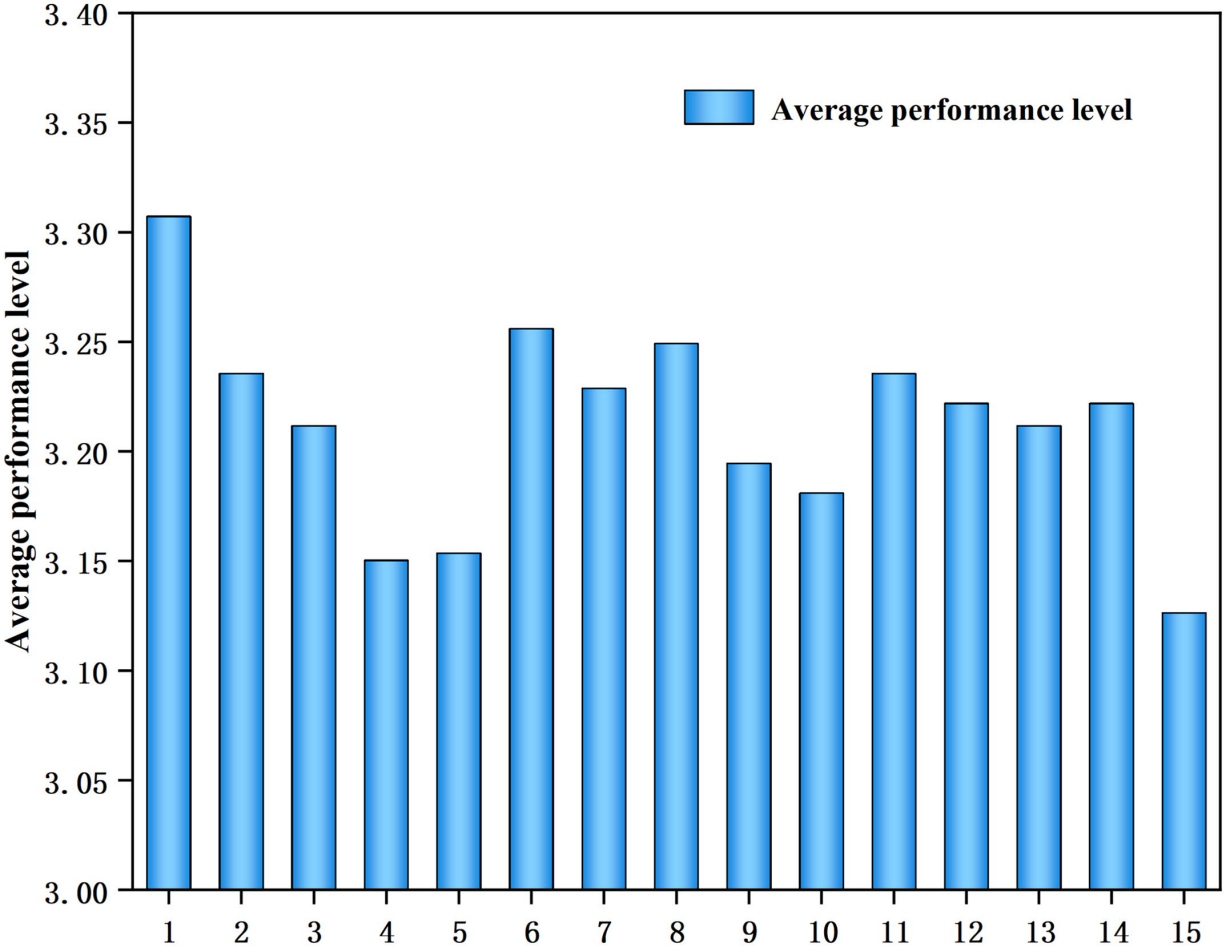
Mean value of the performance level of the ideological and political teaching ability of professional teachers in universities empowered by digital intelligence.

The performance level of teachers’ ideological and political teaching abilities in universities can be assessed using a questionnaire created using the indicators in the inspection model (see Figure 2).

As shown in Figure 2, the mean performance scores for the ideological and political teaching ability of digitally intelligence-empowered college teachers exceeds the mean value of 2.5. The highest mean score was 3.31 (ideological and political literacy ability), and the lowest mean value was 3.13 (guaranteeing ability of the teaching and learning management departments). This indicates that the overall performance level falls within the middle to upper level. This is a result of the great importance placed on cultivating qualified talent in colleges and universities, deepening reforms in ideological and political education, and ideological and political initiatives for improving training for teachers.

#### 4.2.2 Overall evaluation of the training needs of the ideological and political teaching ability of the professional teachers universities empowered by digital intelligence

**Figure 3:**
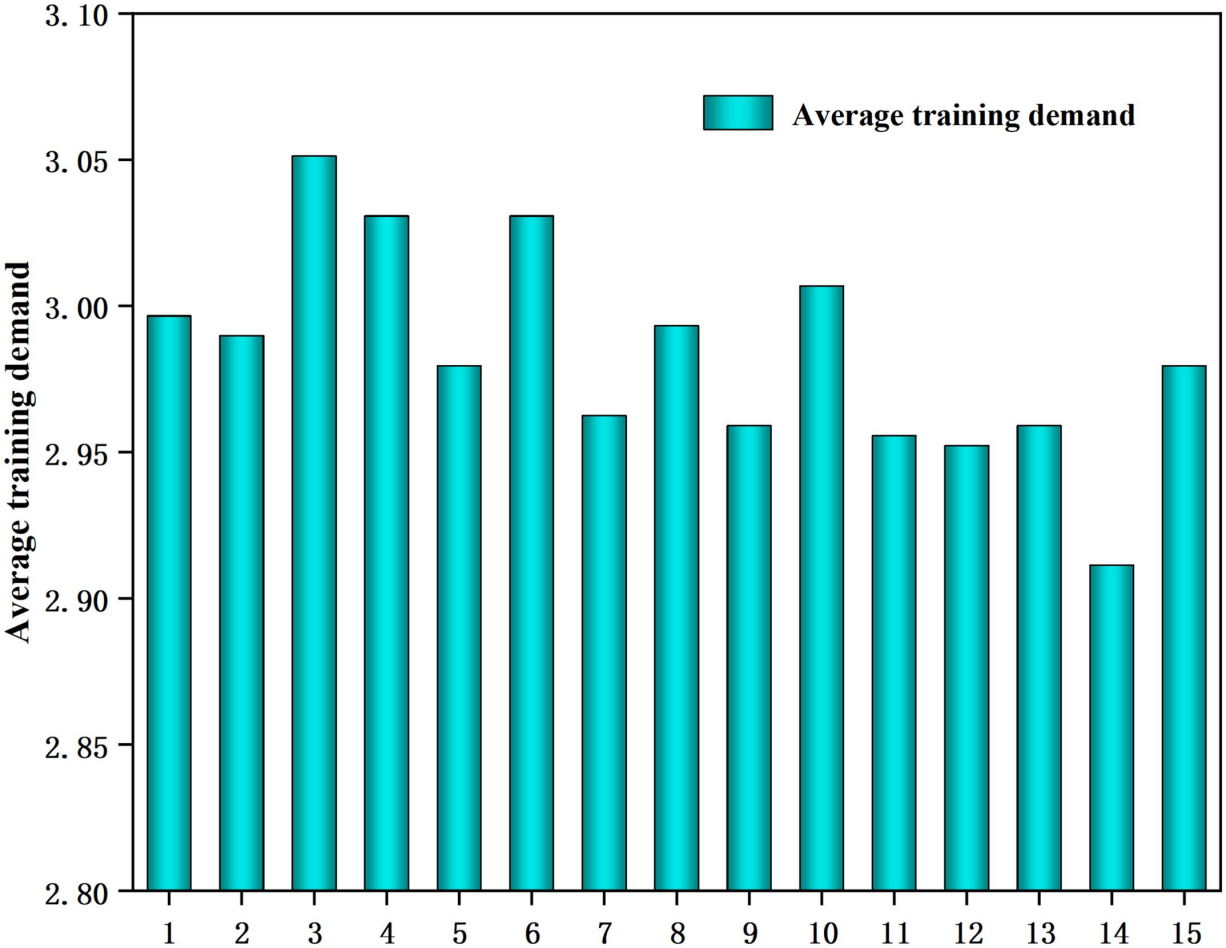
The average of the training needs of ideological and political teaching ability of professional teachers in universities empowered by digital intelligence.

By analyzing the training needs of professional teachers’ ideological and political teaching abilities in digitally empowered colleges and universities, we can reveal the varying degrees of training needs (Figure 3).

As shown in Figure 3, professional instructors in universities equipped with digital intelligence have training demands for political and ideological instruction that are higher than the average of 2.5. The highest average score was 3.05, corresponding to the need to improve teaching methods, innovate pedagogical concepts, and fully explore ideological and political education materials in various ways. The lowest average was 2.91, corresponding to the need for self-reflection to evaluate the actual effect of ideological and political education from the teacher’s own perspective. This indicates that specialized course instructors exhibit a strong demand for training, particularly in areas related to teaching methods, pedagogical concepts, and ideological and political educational material.

A comparative analysis of Figures 2, 3 shows that a lower mean performance does not necessarily entail higher training demand. For example, the average value of “the ability to creatively integrate the educational resources of ideology and politics with the teaching content of the curriculum” ranks the third lowest in performance but sixth lowest in training demand. Similarly, a high average value of performance level does not indicate low training need. For example, “the ability to possess political literacy” had the highest average performance score but ranked fifth in training demand. Before the implementation of formal training programs targeting political thinking ability, we should not formulate a training program based on the results of a one-sided analysis. A comprehensive evaluation of actual demand is necessary to formulate a more suitable training program for teachers of professional courses.

### 4.3 The difference of performance level of ideological and political teaching ability of professional teachers in digital intelligence is analyzed

We employed a one-way ANOVA to investigate differences in the ideological and political teaching proficiency of professional instructors at institutions equipped with digital intelligence. The results are summarized in Table 6.

**Table 6:** Differential analysis of the performance level of ideological and political teaching ability of professional teachers in universities empowered by digital intelligence and universities list.

Question item	Gender		Professional ranks and titles		Educational background		School age	
	*F* value	*p* value	F value	*p* value	F value	*p* value	F value	*p* value
1	1.154	0.284	6.874	0.000^**^	4.648	0.010^*^	1.904	0.110
2	3.493	0.063	10.273	0.000^**^	3.331	0.037^*^	3.474	0.009^**^
3	3.471	0.063	3.115	0.027^*^	2.015	0.135	1.777	0.134
4	4.679	0.031^*^	1.627	0.183	1.364	0.257	1.056	0.379
5	4.445	0.036^*^	1.548	0.202	3.499	0.032^*^	0.593	0.668
6	4.900	0.028^*^	4.012	0.008^**^	1.397	0.249	0.626	0.644
7	2.427	0.120	4.236	0.006^**^	0.699	0.498	1.328	0.260
8	1.135	0.288	3.360	0.019^*^	2.981	0.052	1.129	0.343
9	4.140	0.043^*^	6.049	0.001^**^	0.786	0.457	1.986	0.097
10	5.061	0.025^*^	1.065	0.364	4.963	0.008^**^	1.108	0.353
11	6.774	0.010^**^	4.792	0.003^**^	2.051	0.130	0.859	0.489
12	3.628	0.058	3.986	0.008^**^	2.773	0.064	1.249	0.291
13	4.726	0.031^*^	1.716	0.164	0.663	0.516	0.307	0.873
14	2.658	0.104	1.524	0.208	2.646	0.073	0.454	0.770
15	0.377	0.539	0.806	0.491	2.324	0.100	1.779	0.133

Table 6 shows that there are differences in 7 out of 15 performance items related to political thinking ability among college professional teachers of different genders. Among them, the item “the ability to introduce elements of nurturing and to realize interpersonal interaction between teachers and students inside and outside the classroom” showed a significant difference (*p* < 0.01). In terms of professional titles, 8 out of 15 items showed differences among instructors. Significant differences (*p* < 0.01) were observed in the following areas: “ideological and political literacy ability,” “ideological and political education comprehension and analysis ability,” “ability to understand students comprehensively and to guide them,” “ideological and political education organization and design,” “the ability to combine professional education with ideological and political education and to manage the classroom based on the teaching objectives of knowledge transfer, ability development, and value building,” “the ability to introduce elements of human development,” “the ability to develop a sense of responsibility for the ‘students,” and “own ideological and political evaluation ability.”

Differences among teachers based on professional qualifications were noted in 4 out of 15 items. A statistically significant difference (p < 0.01) was found in “the ability to use modern information technology to enrich and expand teaching resources for ideological and political education, and the ability to utilize modern information technology to realize interoperable blended teaching between on-line and off-line groups.” Regarding teaching experience, a significant difference (p < 0.01) was observed only in the item related to “ability to comprehend and analyze ideological and Political Education.”

### 4.4 Analysis of the differences in the training needs of the professional teachers’ ideological and political teaching ability in universities empowered by digital intelligence

To explore the differences in the training needs for ideological and political teaching ability among teachers in digital empowered universities, a one-way ANOVA was used, and the results are shown in Table 7.

**Table 7:** Differential analysis of training needs of ideological and political teaching ability training needs of professional teachers empowered by digital intelligence.

Question item	Gender		Professional ranks and titles		Educational background		School age	
	F value	*p* value	F value	*p* value	F value	*p* value	F value	*p* value
1	0.526	0.469	0.665	0.574	0.257	0.774	0.455	0.769
2	0.524	0.470	0.604	0.613	0.268	0.765	0.409	0.802
3	1.197	0.275	1.065	0.364	0.068	0.935	1.427	0.225
4	2.214	0.138	0.775	0.509	0.393	0.675	0.863	0.487
5	3.202	0.075	0.658	0.578	0.971	0.380	0.563	0.690
6	1.724	0.190	0.391	0.760	2.324	0.100	0.893	0.468
7	0.985	0.322	2.004	0.114	0.167	0.846	0.730	0.572
8	0.348	0.556	0.839	0.473	0.751	0.473	1.701	0.150
9	1.697	0.194	1.824	0.143	0.514	0.599	1.416	0.229
10	1.986	0.160	0.242	0.867	0.570	0.566	1.277	0.279
11	1.074	0.301	1.439	0.231	0.111	0.895	1.078	0.368
12	2.349	0.126	1.191	0.314	1.283	0.279	1.433	0.223
13	1.718	0.191	1.679	0.172	0.885	0.414	1.648	0.162
14	2.733	0.099	0.992	0.397	0.978	0.377	2.318	0.057
15	4.892	0.028^*^	0.558	0.643	0.604	0.547	1.310	0.266

As seen in Table 7, there were no statistically significant differences in training needs of professional teachers’ based on different titles, qualifications, and years of teaching experience. A gender-based difference was observed in one item, “Safeguarding ability of teaching management departments.” Schools and colleges have evaluation mechanisms to enhance the effectiveness of education through quality assessment systems, title evaluation, teacher development, and incentive mechanisms. A significant difference was observed (*p* < 0.05).

### 4.5 Analysis of performance level and training demand trend of ideological and political teaching ability of college teachers of different genders

**Figure 4:**
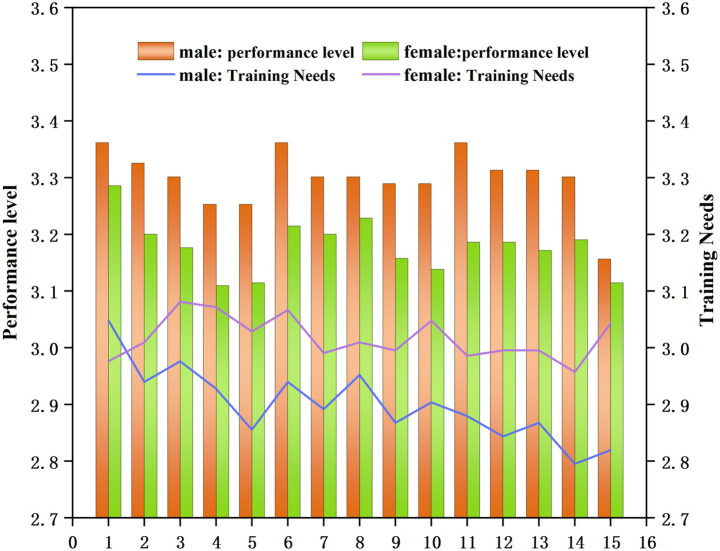
Analysis of gender differences in the performance level and training needs of professional teachers in universities empowered by digital intelligence.

To analyze gender-based trends in both performance level and training demand, an independent samples t-test was conducted using the results from the four-options related to the political teaching ability and corresponding training needs. The results are shown in Figure 4.

As illustrated in Figure 4, male college teachers demonstrated better results in ideological and political ability for all questions. This may be attributed to male teachers’ more logical teaching style and more optimistic attitude toward their performance level.

Regarding training needs, male teachers expressed the strongest need for training in ideological and political literacy. Female teachers showed the highest training needs in continuously improving teaching methods, innovative teaching concepts, and the ability to fully utilize and collect ideological and political educational materials from multiple perspectives.

### 4.6 The performance level of ideological and political teaching ability of professional teachers with different degrees are analyzed

**Figure 5:**
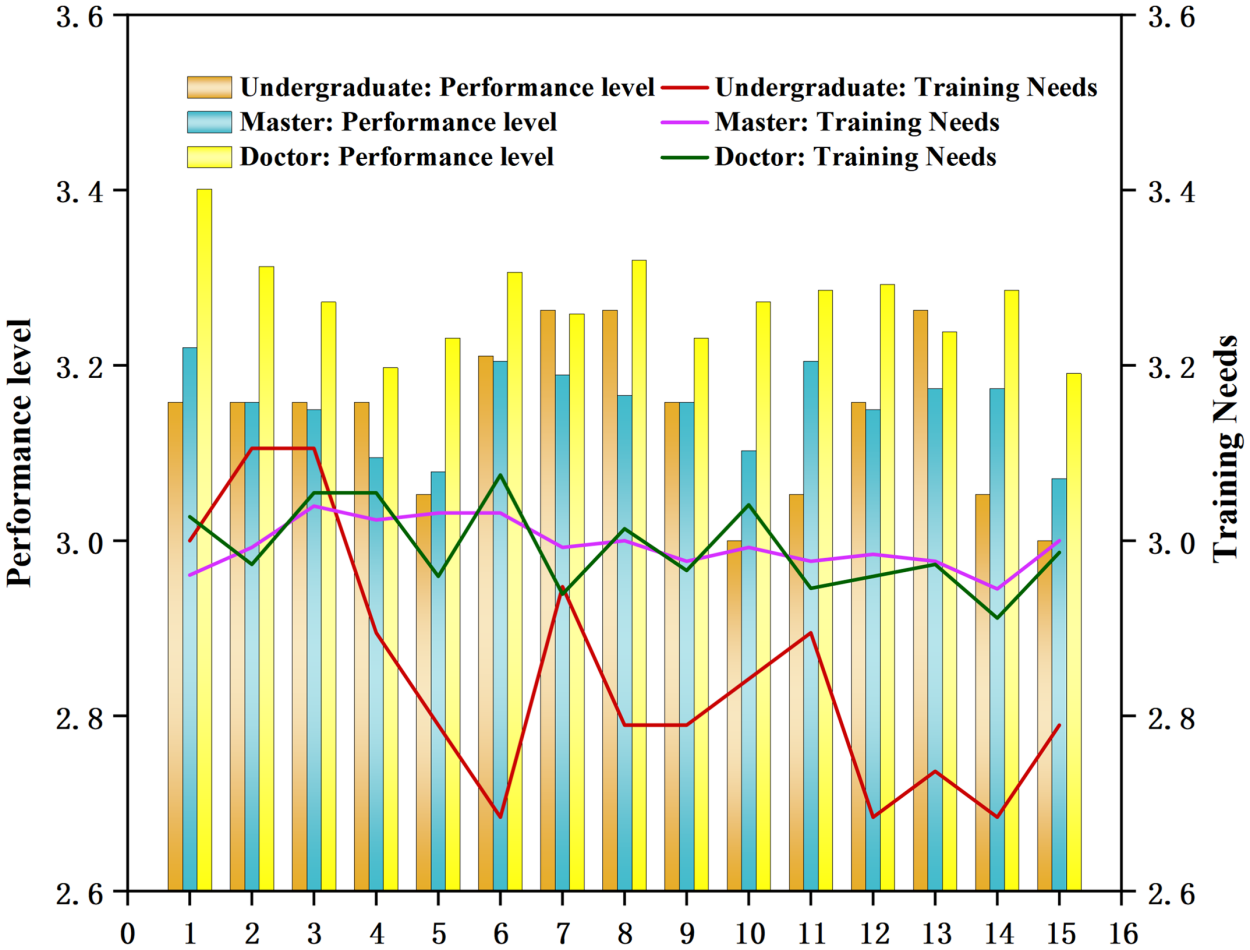
Analysis of the performance level of ideological and political teaching ability and training needs of professional teachers in universities empowered by digital intelligence.

Using the independent sample t-test, we analyzed responses to the four-option scale in the questionnaire completed by professional teachers with different academic degrees. The results are shown in Figure 5.

As shown in Figure 5, teachers with doctoral degrees consistently outperform those with other degrees in the performance level of ideological and political teaching ability in digitally intelligence-empowered colleges and universities. Teachers with bachelor’s degrees perform significantly lower than those with master’s and doctoral degrees in this area. Regarding training needs related to ideological and political teaching in digitally empowered environments, teachers with master’s and doctoral degrees showed strong and comparable levels of training demand. However, teachers with bachelor’s degrees exhibited fluctuating and significantly lower training needs. As the figure shows, higher educational level is associated with both stronger teaching performance and greater training needs in ideological and political education. The bachelor’s degree teachers with lower performance levels also displayed lower training needs.

Therefore, from a professional development standpoint, colleges and universities should provide selective development opportunities for teachers with high academic qualifications. Lower-educated undergraduate teachers should be allowed to re-examine themselves, refine the points of poor performance, and carry out training efforts.

### 4.7 The performance level of ideological and political teaching ability of different professional teachers are analyzed

**Figure 6:**
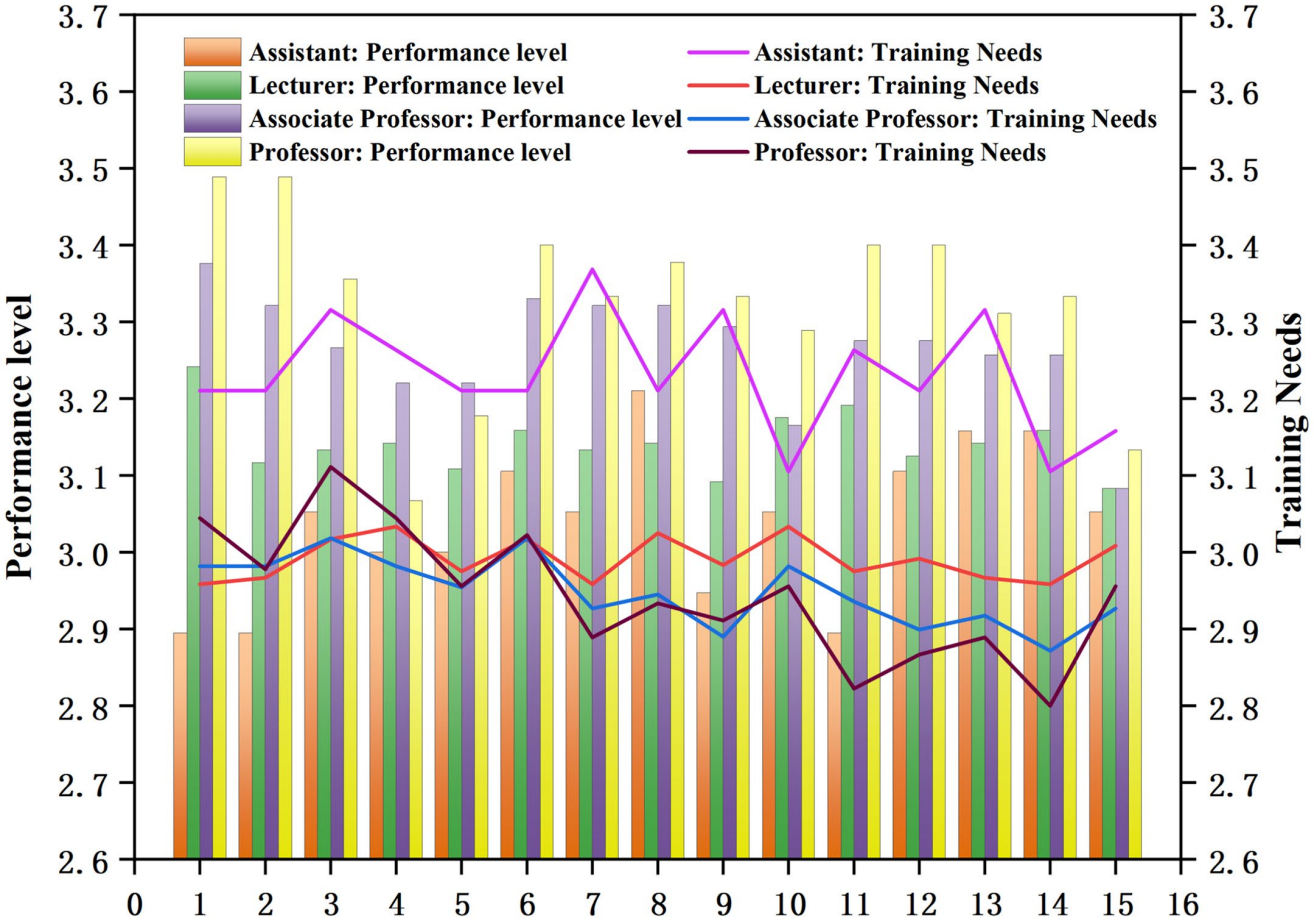
Analysis of the title difference of ideological and political teaching ability performance level and training demand of professional teachers in universities empowered by digital intelligence.

Using the independent sample t-test, we analyzed the questionnaire responses on ideological and political teaching ability among college teachers with different academic titles. The results are shown in Figure 6.

As shown in Figure 6, the performance level of ideological and political teaching ability increases with academic rank: assistant professors (junior) demonstrated lowest performance levels, while full professors (senior) showed the highest. Among the 12, 13, and 14 questions, the college teachers of all academic ranks showed excellent and balanced performance levels, which assess “their own ability to evaluate the ideology of politics,” “the influence of collaborative cultivation of human beings, to influence the ideology and cultural literacy of students, actively participate in activities, and to produce the performance, emotions, attitudes and behaviors of the students. Performance, emotions, attitudes, and behavioral changes,” and “self-reflective ability to assess the actual effectiveness of ideologies and politics teaching from the teacher’s own point of view.”

Regarding training needs for ideological and political teaching ability in digitally empowered environments, assistant professors demonstrated strong demand across all topics. In contrast, training needs among professors fluctuated greatly by topics and showed the lowest average training demand. Among these, training needs were high for these two items: “the ability to continuously improve teaching methods, innovate teaching concepts, and fully explore and collect materials for ideological and political education from various sources” and “the ability to effectively package the elements of ideologies and politics with the help of digital technology and current political hotspots.” Therefore, there is a need to provide targeted training on specific competencies, and strengthen the training of assistants professors.

### 4.8 Analyzing the trend of the performance level and training needs of the ideological and political teaching ability of university professional teachers of different teaching ages empowered by numerical intelligence

**Figure 7:**
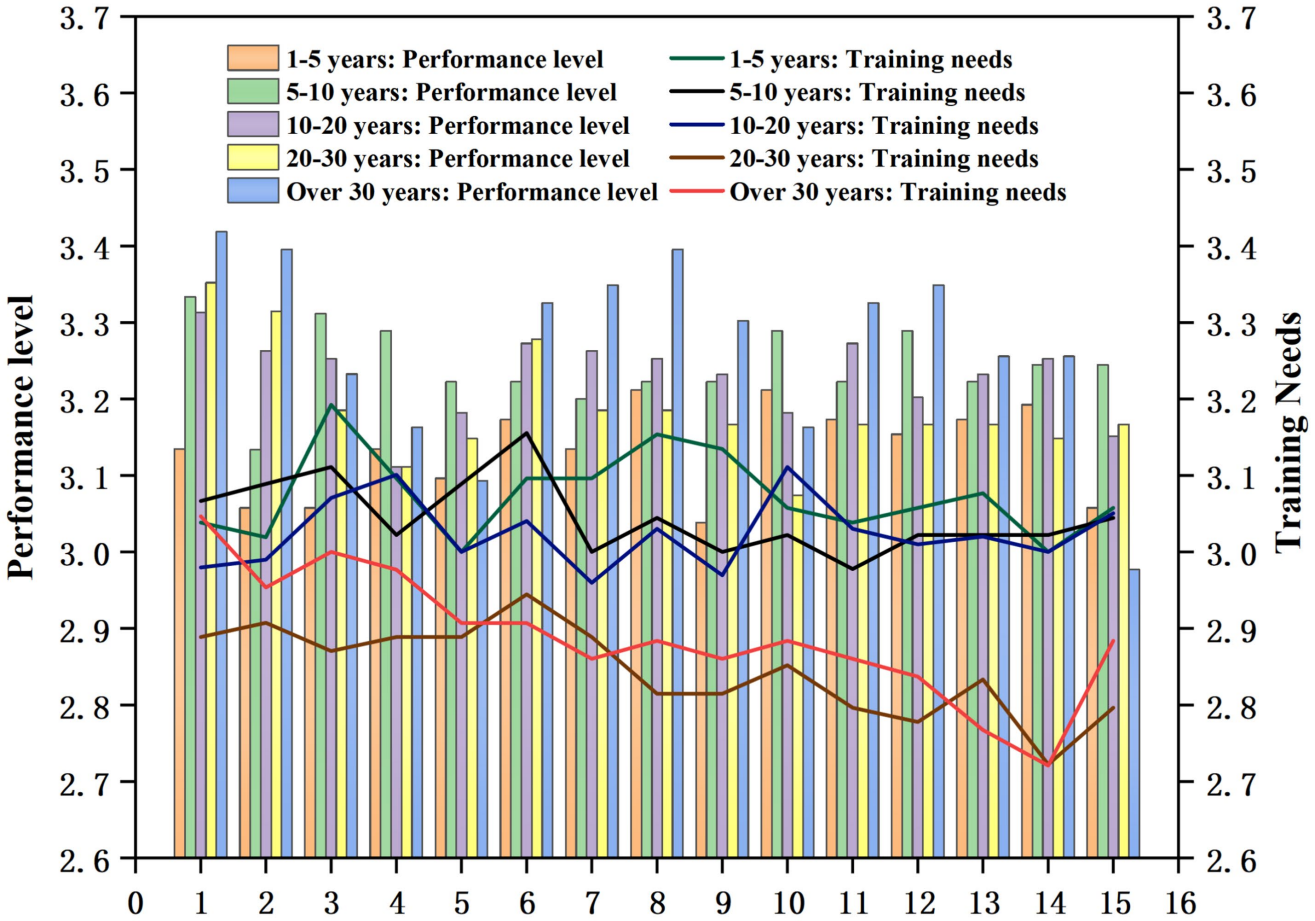
Analysis of teaching experience differences in the performance level and training needs of professional teachers’ ideologies and politics teaching ability in universities empowered by digital intelligence.

The independent samples t-test was used to analyze the four-option questionnaire items on both the performance level and training needs of ideological and political teaching ability among professional college teachers with different teaching tenures. The results are shown in Figure 7.

As can be seen in Figure 7, in ideologically and politically empowered environments, teachers with 1–5 years of teaching experience generally performed poorly, whereas teachers with 30 years of teaching experience or more performed excellently in following areas: “ability to possess ideological and political literacy,” “the ability to organize and design ideological and political teaching,” “the ability to integrate elements of political identity, national sentiment, and social responsibility into professional classroom teaching,” and “Ability to fully understand and guide students.”

In terms of training demand, teachers with 1–5, 5–10, and 10–20 years of teaching experience exhibited generally high demand, with the strongest training demand observed among teachers with 1–5 years of teaching experience. The highest-rated training item was “the ability to continuously improve teaching methods, innovate the concept of teaching, and to fully excavate and collect ideologies and politics educational materials.”

Thus, it can be seen that there is a positive correlation between teaching experience and the performance level of ideological and political teaching ability. Conversely, less experienced teachers demanded stronger training needs.

## 5 Originality and potential influence of the model for checking the ideological and political teaching ability of teachers in colleges and universities empowered by digital intelligence

### 5.1 Comparison and innovation with UNESCO Global Citizenship Education Framework

The digital-intelligence-empowered model for assessing the ideological and political teaching ability of professional college teachers, as constructed in this study, is in sharp contrast to the UNESCO Global Citizenship Education Framework (GCED) in terms of target positioning, core elements, technology application, and international adaptability, reflecting the significant advantages of China’s ideological and political models and technology integration, as shown in Table 8.

**Table 8:** Comparison between the ideological and political teaching ability inspection model and UNESCO GCED framework.

Compare dimensions	UNESCO Global Citizenship Education Framework (GCED)	Model for ideological and political teaching evaluation	Originality analysis
Target location	Focus on global citizenship and cross-cultural understanding	Focuses on ideological and political communication and teaching ability of Chinese college teachers	Transforms the universality of global education into a nationally oriented ideological and political model
Core elements	Emphasis on knowledge integration, ability cultivation, and value shaping	Emphasizes ideological, political education, and technology-driven accurate evaluation	Creatively integrates digital tools, political themes, and course content in the classroom
Technology application	Advocates experimental application of open educational resources and virtual reality technology	Applies big data analytics and artificial intelligence dynamic mapping	Technology serves the precision of ideological and political education
International adaptability	Offers universal applicability but lacks in-depth exploration of the ideological and political field in China	Integrates GCED concepts with the ideological and political situation in China	Realizes the localization of international experience

### 5.2 The potential impact of intelligent digitalization on the verification model of ideological and political teaching ability of teachers in colleges and universities

The digital intelligence-empowered model for assessing the ideological and political teaching ability of college teachers developed in this study shows unique practical value and theoretical contribution at the international level through the approach of “technology empowerment + localization innovation.” The model is based on five core elements: 1. ideological and political literacy and cognitive abilities, 2. exploration and development capabilities, 3. organizational and design capabilities, 4. implementation and innovation capabilities, and 5. evaluation and support capabilities, alongside 15 capability indicators. It constructs a dynamic closed-loop assessment mechanism through a mixed research method that includes literature coding, behavioral event interviews, the Delphi method, and questionnaire surveys (Cronbach’s alpha > 0.8 and confirmatory factor analysis fit indices meeting established standards). The model achieves precise implementation of ideological and political education through artificial intelligence dynamic graphs and big data analysis techniques. By leveraging the TPACK framework, the model shifts ideological and political education from “formal indoctrination” to “subtle infiltration, like salt dissolving in water.” This model can provide new insights to enhance the UNESCO Global Citizenship Education Framework by addressing the issue of uneven distribution of national educational resources. Its scientific rigor is validated through high reliability and validity by integrating “digital tools + localization indicators,” combining “ideological and political capabilities” with “technological governance.” This study presents a case study for a globally applicable education assessment framework that merges ideological and political education with technological empowerment. Dong et al. (2023) propose constructing a “three-stage all-encompassing atmosphere” network for ideological and political education using data mining and artificial intelligence technologies, optimizing the course environment and addressing the timeliness issue in ideological and political education (Dong et al., 2023). This article emphasizes the use of digital technology to develop shared ideological and political education course resources, with the aim of enhancing the ability of professional teachers in higher education institutions to explore and develop such courses. Both emphasize the necessity of integrating technology for the development of ideological and political education resources; however, this study further proposes a tiered training strategy and a “collaborative research community” mechanism, addressing the shortcomings in resource allocation and teacher capability differences identified in previous studies against the background of digital, ideological, and political education transformation.

Du and Cao (2023) used intelligent image recognition algorithms to analyze classroom teacher-student behavior, verifying the effectiveness of artificial intelligence in assessing teaching engagement and outcomes through 95% behavioral recognition accuracy (Du and Cao, 2023). This paper, through questionnaire surveys and reliability validation (Cronbach’s Alpha > 0.8, CFI = 0.952), suggests that teachers should receive targeted training in “integration of ideological and political resources” and “ideological and political literacy and cognitive abilities.” While both studies rely on quantitative tools (AI behavioral recognition models/statistical models) to assess teaching capabilities, this study particularly focuses on differences in teacher competency needs (gender, teaching experience, and title), providing data to support the precise training of teachers in the context of digital, ideological, and political education transformation.

## 6 Conclusions and implications of the study

Universities are placing increasingly high requirements for the integration of teachers’ ideological education and professional education. This study constructs a model framework for inspecting the ideological and political teaching abilities of professional teachers, incorporating five basic elements and 15 ability index points. Cronbach’s alpha value for the five elements all exceeded 0.8, indicating strong internal consistency and reliability. The KMO value was 0.950, which was greater than 0.7. The chi-square value of the Bartlett's test was 3,314. The test had a chi-square value of 3314.006, degrees of freedom of 105, and a *p*-value of 0.00, indicating that the research data were suitable for extracting information. Factor extraction using principal component analysis with five common factors yielded a satisfactory explanation of 79.182% > 50% of the cumulative variance after rotation.

The 15 indicators in the digital-intelligence-empowered evaluation model showed minimum loadings of 0.532 with no overlap, and each of the five factors corresponded clearly with the model’s five dimensions. The standardized factor loadings for all ability elements exceeded 0.5 and have a strong correlation. The CR values of the essentials were all greater than 0.8, above the threshold of 0.6. The AVE values of the mean extraction variance exceeded 0.5, confirming high convergent and discriminant validity.

Furthermore, the inspection model was used to evaluate the performance level of university teachers in ideological and political teaching abilities and the training needs of teachers, empowered by digital intelligence, resulting in three key insights:

- The demand for training in ideological and political education is high. Teachers generally express a strong willingness to learn, but lack the ability to explore and develop them. Training should focus on improving teaching methods, innovating teaching concepts, fully excavating and collecting the technical ability of ideological and political education materials from various sources, strengthening how to utilize digital technology, understanding current political hotspots, and effectively using political elements to enhance ideological and political education development.
- The high-quality development of ideological and political teaching abilities requires establishing a “cooperative research community” for ideology and political education. The community should comprise teachers of ideology and politics courses, teachers of professional courses, teaching management departments, student counselors, and so on, to build a “professional ideology and politics education teaching resource base.” With the help of digital technology, we can develop targeted and high-quality sharing and crowdfunding of resources to improve the ideological and political teaching abilities and ideological literacy of professional course teachers.
- To optimize the evaluation and support mechanisms for ideological and political education, a multi-level, multi-dimensional, and comprehensive mechanism is necessary. By linking the evaluation of the performance into annual reviews and awards, title evaluation, recruitment, promotion, etc., we can enhance the initiative and willingness of the ideological and political education of professional teachers in terms of institutional guarantee. Moreover, a broader support system involving educational authorities, schools, and colleges should be established to ensure robust quality assurance, faculty development, and incentive mechanisms to strengthen ideological and political education.

### 6.1 Analysis of research limitations

Bias in self-reporting: Data for this model were collected through questionnaires. Although the questionnaire design was rigorous, the responses provided by teachers were biased toward their own subjective perceptions, and some may have overestimated themselves. Additionally, some teachers were also influenced by social expectations, leading to a bias in the results.

*Limitations of the cross-sectional design*: The data for this model were collected at a single time point, and questionnaire distribution and collection took time. During this period, teachers’ abilities may change, which this study failed to control for. Consequently, the model may have overestimated or underestimated the dynamic changes in teachers’ actual abilities.

*Geographical scope limitation*: Due to the research team’s faculty resources being limited to Changchun, Tianjin, and Chongqing, the sample includes only nine universities (both key and local) with different majors in these three places; therefore, the applicability of the model may be limited.

*Lack of external validation*: The model validation is currently based only on local Chinese data and has not been tested in other countries or education systems.

To overcome these limitations, in future research we will not only incorporate more objective statements to the questionnaire but also adopt a cross-investigation approach for teachers from different disciplines to reduce the subjective judgment of expert teachers. We will conduct long-term tracking studies, evaluating the same group of teachers annually to observe changes in their “ideological and political literacy” indicators. We will expand our reach to northeast, north, and southwest China to test the robustness of the model under different economic levels and cultural backgrounds. On this basis, we will pilot collaborations with overseas universities in countries along the “Belt and Road” route (such as Malaysia and Egypt) and adjust the localization indicators in the model to test international adaptability. We aim to collaborate with international organizations to build a data-sharing platform, work with universities in different countries to modify the indicators in the model according to their cultural characteristics, collect feedback from actual use, and continuously adjust the model to better meet the needs of various countries.

## Data Availability

The original contributions presented in the study are included in the article/supplementary material, further inquiries can be directed to the corresponding author.

## References

[ref1] Akour M. Alenezi M. (2022). Higher education future in the era of digital transformation. Educ. Sci. 12:784. doi: 10.3390/educsci12110784

[ref2] Biggs M. (2003). Positive feedback in collective mobilization: the American strike wave of 1886. Theory Soc. 32, 217–254. doi: 10.1023/A:1023905019461

[ref3] Bygstad B. Øvrelid E. Ludvigsen S. Dæhlen M. (2022). From dual digitalization to digital learning space: exploring the digital transformation of higher education. Comput. Educ. 182:104463. doi: 10.1016/j.compedu.2022.104463

[ref4] Chen J. (2023). Research on ideological and political teaching of college English course under the POA theory. Int. J. Educ. Humanit. 11, 545–547. doi: 10.54097/ijeh.v11i3.15168

[ref5] de Fátima Cruz M. Franco M. Rodrigues M. (2022). Exploring university–firm collaboration in teaching: a case study of co-creation. Ind. Higher Educ. 36, 667–679. doi: 10.1177/09504222211049347

[ref6] Dong F. Dong S. Xue J. (2023). “Research on the optimization of ideological and political education in universities integrating artificial intelligence technology under the guidance of curriculum ideological and political thinking,’’ in *ACM Transactions on Asian and Low-Resource Language Information Processing*.

[ref7] Du J. Cao J. (2023). Exploration of the ideological and political elements of artificial intelligence courses under the background of three comprehensive education. Wirel. Commun. Mob. Comput. 2023, 1–11. doi: 10.1155/2023/2427039, PMID: 40860520

[ref8] Freeman D. Reeve S. Robinson A. Ehlers A. Clark D. Spanlang B. . (2017). Virtual reality in the assessment, understanding, and treatment of mental health disorders. Psychol. Med. 47, 2393–2400. doi: 10.1017/S003329171700040X, PMID: 28325167 PMC5964457

[ref9] Freire P. (1970). Cultural action and conscientization. Harvard Educ Rev. 40, 452–477. doi: 10.17763/haer.40.3.h76250x720j43175

[ref10] Honglan L. Wu R. (2021). Study on problems and countermeasures of ideological and political teaching in colleges and universities under the background of new media era. J. Intell. Fuzzy Syst., 1–9. doi: 10.3233/JIFS-219139, PMID: 39743787

[ref11] Hongmei W. Songlin T. (2021). Sentiment analysis of students in ideological and political teaching based on artificial intelligence and data mining. J. Intell. Fuzzy Syst., 1–10. doi: 10.3233/JIFS-219047, PMID: 39743787

[ref12] Howard M. C. (2017). A meta-analysis and systematic literature review of virtual reality rehabilitation programs. Comput. Hum. Behav. 70, 317–327. doi: 10.1016/j.chb.2017.01.013

[ref13] Jiao Y. Liu Y. Gupta P. (2021). The teaching optimization algorithm mode of integrating mobile cloud teaching into ideological and political courses under the internet thinking mode. Sci. Program. 1, 1–8. doi: 10.1155/2021/6492009

[ref14] Liu Y. Jia T. (2023). Research on the pattern of college curriculum education based on curriculum ideology and politics in the new era. J. Educ. Educ. Res. 1, 73–76. doi: 10.54097/jeer.v1i3.4802

[ref15] Luo J. (2020). Teaching reform of ideological and political courses based on “internet+”. J. Phys. Conf. Series 1553:2020. doi: 10.1088/1742-6596/1533/4/042020

[ref16] Makransky G. Terkildsen T. S. Mayer R. E. (2019). Adding immersive virtual reality to a science lab simulation causes more presence but less learning. Learn. Instruction. 60, 225–236. doi: 10.1016/j.learninstruc.2017.12.007

[ref17] Mezirow J. (1992). Transformation theory: critique and confusion. Adult Educ Quart 42, 250–252. doi: 10.1177/074171369204200404

[ref18] Mishra P. Koehler M. J. (2006). Technological Pedagogical content knowledge: A framework for teacher knowledge. Teach Coll Rec. 108, 1017–1054. doi: 10.1177/016146810610800610

[ref19] Moreira P. D. L. Neto J. R. Sabucedo J. M. Camino C. P. D. S. (2018). Moral judgment, political ideology and collective action. Scand. J. Psychol. 59, 610–620. doi: 10.1111/sjop.12479, PMID: 30091786

[ref20] Pereira R. Franco M. (2023). University-firm cooperation and regional development: proposal of a model of analysis. J. Knowl. Econ. 14, 676–690. doi: 10.1007/s13132-022-00947-6

[ref21] Rawal D. M. (2024). Mapping of school teachers’ digital competency in the context of digital infrastructure: a systematic review and empirical study of India. J. Prof. Cap. Community. 9, 173–195. doi: 10.1108/JPCC-01-2024-0016, PMID: 35579975

[ref22] Shao D. (2023). The dilemma and promotion strategy of ideological and political construction of university curriculum. J. Educ. Educ. Res. 2, 42–46. doi: 10.54097/jeer.v2i1.5220

[ref23] Shulman G. (1986). Gerrard Winstanley: The radicalism of the good son. Polity. 18, 473–497. doi: 10.2307/3234771

[ref24] Tatto M. T. (2023). Developing teachers’ research capacity: the essential role of teacher education. Teach Educ 32, 1–20. doi: 10.1080/10476210.2020.1860000

[ref25] Väisänen S. Hirsto L. (2020). How can flipped classroom approach support the development of university students’ working life skills?—university teachers’ viewpoint. Educ. Sci. 10:366. doi: 10.3390/educsci10120366

[ref26] Wang M. (2022). Design and practice of ideological and political teaching of database principle and application course. SHS Web Conf. 145:1026. doi: 10.1051/shsconf/202214501026

[ref27] Wang F. (2024). Exploration of cultural teaching mechanisms of ideology and politics courses under the background of “internet plus”. Appl. Math. Nonlinear Sci. 9:1407. doi: 10.2478/amns.2023.2.01407, PMID: 40529641

[ref28] Wang Q.-L. Liu L.-L. Liu C.-R. Zhu Q.-S. Ren Z.-Y. Gang T.-T. . (2023). ‘Internet+’ comprehensive nursing training course in the post-epidemic era-an exploration of the mixed teaching mode: a randomized trial. Front. Med. 10:1152732. doi: 10.3389/fmed.2023.1152732, PMID: 37448807 PMC10336544

[ref29] Watfa M. K. Audi D. (2017). Innovative virtual and collaborative teaching methodologies. Behav. Inf. Technol. 36, 663–673. doi: 10.1080/0144929X.2016.1275806

[ref30] Zhao C. Yu J. (2024). Relationship between teacher’s ability model and students’ behavior based on emotion-behavior relevance theory and artificial intelligence technology under the background of curriculum ideological and political education. Learn. Motiv. 88:102040. doi: 10.1016/j.lmot.2024.102040

[ref31] Zheng P. Yang J. Lou J. Wang B. (2024). Design and application of virtual simulation teaching platform for intelligent manufacturing. Sci. Rep. 14:12895. doi: 10.1038/s41598-024-62072-5, PMID: 38839812 PMC11153506

[ref32] Zhu Y. Zheng L. (2021). Ideological and political teaching information management based on artificial intelligence and data security model. J. Intell. Fuzzy Syst., 1–11. doi: 10.3233/JIFS-219126, PMID: 39743787

